# Does Preoperative Coffee Consumption Prevent Intraoperative Hypotension in Cesarean Section Surgeries?

**DOI:** 10.7759/cureus.63584

**Published:** 2024-07-01

**Authors:** Gamze Talih, Ayşe Ulgey, Merve Şahingöz, Fatma Özdemir, Kudret Dogru, Aliye Esmaoğlu

**Affiliations:** 1 Anesthesiology and Reanimation, Erciyes University Faculty of Medicine, Kayseri, TUR; 2 Anesthesia and Critical Care, Erciyes University Faculty of Medicine, Kayseri, TUR; 3 Anesthesiology and Reanimation, Kırşehir Education and Research Hospital, Kırşehir, TUR; 4 Obstetrics and Gynecology, Erciyes University Faculty of Medicine, Kayseri, TUR

**Keywords:** post-dural puncture headache, spinal anaesthesia, post-spinal hypotension, coffee, cesarean section

## Abstract

Objective: In this study, we evaluated the effects of a cup of coffee given to patients before surgery in a cesarean section by means of intraoperative hypotension, ephedrine requirement, and the incidence of post-dural puncture headache (PDPH).

Methods: A total of 140 patients undergoing elective cesarean section with spinal anesthesia were included in this study. Participants who drank a single cup of filtered coffee two hours before spinal anesthesia were included in the coffee group, and those who drank water were in the control group. In each group, 70 patients were included. Hemodynamic parameters were recorded every three to five minutes after spinal anesthesia. Intraoperative use of ephedrine was recorded. The PDPH was monitored for three days.

Results: The incidence of intraoperative hypotension was 48.6% in the coffee group and 71.4% in the control group (p = 0.006). The rate of ephedrine usage (25.7%) was significantly lower in the coffee group (p = 0.001). The incidence of PDPH in the first 24 hours (2.9%) was significantly lower in the coffee group (11.4%). The visual analog scale (VAS) score was similar between groups (p = 0.048, p > 0.05).

Conclusions: Consumption of a single cup of coffee before spinal anesthesia reduced the incidence of intraoperative hypotension and the rate of ephedrine usage in cesarean sections.

## Introduction

Spinal anesthesia for a cesarean section is a popular, practical, and safe anesthesia technique. Post-spinal hypotension and post-dural puncture headache (PDPH) are common complications of spinal anesthesia [1]. Hypotension after spinal anesthesia is an important clinical issue that occurs more frequently in obstetric patients, in up to 70% of cases, during surgical procedures [2]. Along with hypotension, nausea, vomiting, bradycardia, changes in consciousness, and even cardiac arrest might occur after spinal anesthesia. Besides the effects observed in the mother, hypotension also has fetal effects. Uteroplacental hypoperfusion, fetal acidosis, asphyxia, and bradycardia might also be observed. Deviation of the uterus to the left, preloading or co-loading of crystalloid or colloid infusion, and using vasopressors such as ephedrine and phenylephrine are some of the various practices that are used to prevent these complications. Phenylephrine has similar effects to ephedrine in the treatment of hypotension; it is associated with less fetal acidosis, nausea, and vomiting [3,4].

While PDPH can occur in the first few hours after spinal anesthesia, in 90% of cases, it occurs in the first three days [5]. It may be severe enough to require an epidural blood patch. It might complicate the mother's baby care and delay her establishment of a healthy bond with the infant [6]. Its frequency ranges from 0.3% to 40%. A lot of factors are affecting the frequency of PDPH. Furthermore, high estrogen levels during pregnancy affect cerebral vascular tone, which makes pregnant women a risky group for PDPH [7]. The incidence of PDPH was found to be 23.47% in a meta-analysis conducted in 2021, which examined eight studies conducted with 175,652 pregnant women [8].

The antioxidant and anti-inflammatory compounds contained in coffee provide cardiovascular protective effects. Coffee has also been shown to have positive effects on neurocognitive dysfunction and is known to increase blood pressure via a cardiostimulant effect where there is no chronic consumption [9].

The use of caffeine in the treatment of PDPH was first reported in 1949. A cup of coffee contains about 100 mg of caffeine. The use of caffeine doses ranging from 75 to 500 mg has been investigated for the treatment of PDPH. The enteral absorption of coffee after oral intake is fast and reaches a peak level in about 30 minutes. Coffee has been reported to reduce PDPH by stimulating the central nervous system and producing cerebral vasoconstriction, and it is successful in headache control [1,10]. In previous studies about caffeine consumption and obstetric surgery procedures, caffeine was used postoperatively [1,6,10]. In our study, we evaluated the effects of preoperative caffeine intake.

The primary aim of this study was to evaluate the incidence of intraoperative post-spinal hypotension and the rate of ephedrine use. The secondary aim was to assess the incidence of PDPH.

## Materials and methods

Study design

Approval was obtained from the Ethics Committee of Erciyes University (Decision No.: 2021/825). A total of 140 pregnant women undergoing elective cesarean section under spinal anesthesia were planned for inclusion in the study after their written consent was obtained. The study was designed following the tenets of the Declaration of Helsinki and was registered at ClinicalTrials.gov (ID: NCT05262933).

Participants

Participants were not randomized for this study. Patients were asked to make a choice among the options presented. In our clinic, we encourage clear fluid intake in patients up to two hours before surgery. A total of 70 pregnant women who preferred to drink filtered coffee (Jacobs Monarch, Brazil) without any sugar were included in the coffee group, and 70 pregnant women who preferred to drink water were included in the control group. Filtered coffee was prepared in a coffee machine. About one teaspoon of coffee was brewed with 150-200 ml of water. It was ensured that coffee was free from particles. About two hours before surgery, patients were asked to drink 150-200 ml (approximately 120 mg of caffeine) of filtered coffee slowly. The patients in the control group were given water in the same amount.

**Figure 1:**
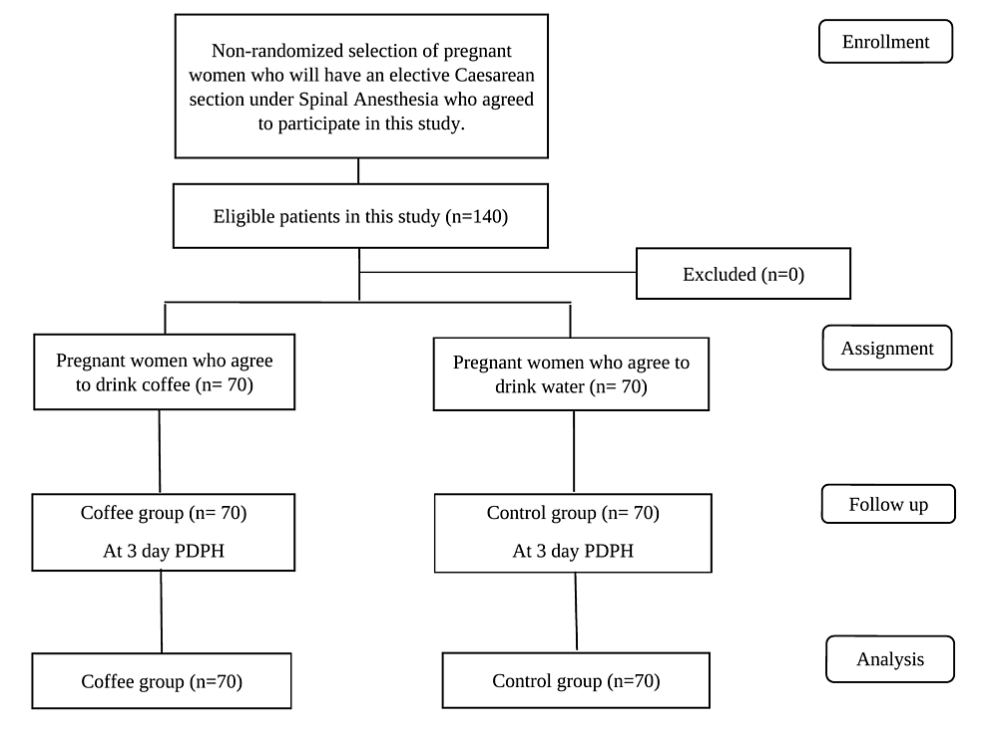
A flow diagram for the non-randomized clinical trial. PDPH: post-dural puncture headache.

Patients who consumed three cups or more of coffee daily, had a coffee or caffeine allergy, had known cardiovascular disease (such as cardiac arrhythmia), cerebrovascular disease, neurological, neurodegenerative or psychiatric disease, diabetes mellitus, or liver disease, patients with pre-eclampsia-eclampsia, and those who had multiple pregnancies were excluded from the study (Figure 1).

Study variables

No premedication was applied to the patients. Patients’ demographic data such as age, gender, height, and weight were recorded. Standard monitoring was performed on the patients in the operating room. The physician who performed the spinal anesthesia was blinded to the study group. Before spinal anesthesia, basal heart rate, non-invasive blood pressure, mean arterial pressure (MAP), and peripheral oxygen saturation (SpO2) were measured and recorded during surgery. Hemodynamic parameters were recorded with an interval of three minutes in the first 15 minutes and subsequent measurements with an interval of five minutes. More than a 20% decrease in baseline MAP value was considered as hypotension and was planned to be treated with 5-10 mg of ephedrine intravenously (IV). In the case of bradycardia (heart rate < 45 beats/min), an IV application of 0.5 mg atropine was planned. A second intravenous cannula (18-20 G) was placed in all patients in the operating room. Isolate-S and 0.9% saline infusions were co-loaded at a volume of 10-15 ml/kg/hour during procedures. Spinal anesthesia was performed with 2.1 ml of 0.5% hyperbaric bupivacaine at the level of L4-L5 or L5-S1 vertebral level in the sitting position with a 25 G Quincke spinal needle from the midline. A dural puncture was performed with the needle direction parallel to the dural fiber in each patient. The patients were placed in a supine position after a surgical sponge was applied over the needle insertion site. The surgical incision was allowed when the sensory block level reached T6. One mg of granisetron was administered intravenously in the case of persistent nausea and vomiting. Besides the data on patient demographics, anesthesia, and surgery duration, the total amount of fluid given, duration of the delivery, 1st minute APGAR (appearance, pulse, grimace, activity, and respiration) scores, total intraoperative rate of ephedrine use, antiemetic use, and presence of nausea and vomiting were recorded. Postoperative pain control was achieved in all patients with dexketoprofen trometamol 50 mg every 12 hours and paracetamol 15 mg/kg every eight hours, which were administered intravenously. Tramadol hydrochloride 1 mg/kg was administered intravenously as the rescue analgesic.

The patients were monitored for three days for post-spinal headaches and questioned about the symptoms of PDPH by an experienced anesthesiologist. After postoperative monitoring of the patients in the hospital for the first 24 hours, the presence of PDPH was questioned by making phone calls to the patients on the second and third days, and their pain scores were recorded using a numerical rating scale (NRS; 0: no pain, 10: worst possible pain). Patients who had PDPH and whose pain has been persistent (NRS > 4) despite conservative treatment (oral hydration, bed rest, caffeine consumption) were advised to take a rescue analgesic (acetaminophen 250 mg + propyphenazone 150 mg + caffeine - Minoset Plus® 500 mg tablet) every six hours in addition to conservative treatment. In the presence of severe PDPH (NRS > 6), which lasted longer than three days despite this treatment, an epidural blood patch was planned for the patient. Post-spinal hypotension and PDPH evaluations were performed by the anesthesiologist who was blind to the study.

Statistical analysis

The number of patients to be included in the study was determined by performing power analysis using similar studies as a reference [3,11]. Accordingly, when an alpha value is set at 0.05 and the power value is 90%, there should be at least 70 patients in each group. The data were statistically analyzed using SPSS version 22.0 (IBM Corp., Armonk, NY). While the mean ± standard deviation (SD) was used for all the continuous variables, frequency and percentage were used for the categorical variables. For normally distributed quantitative data, an independent-sample t-test, which is a parametric test, was performed. Chi-square (χ2) and Fisher’s exact tests were carried out for categorical variables, and a value of p < 0.05 was accepted as statistically significant.

## Results

Data from a total of 140 patients, 70 in each group, were analyzed. There was no statistical difference between the groups when the age, height, weight, duration of anesthesia and surgery, and the total amount of intraoperative fluid given were compared. The mean APGAR scores of the groups were similar (p > 0.05; Table 1).

**Table 1:** Comparison of demographic and perioperative data of the groups. All data are stated as mean ± SD. p < 0.05 according to the independent sample t-test. APGAR: appearance, pulse, grimace, activity, and respiration.

Variables	Control group (n = 70)	Coffee group (n = 70)	p
Age (years)	30.11 ± 6.47	30.50 ± 5.93	0.714
Weight (kg)	81.18 ± 13.60	81.14 ± 11.91	0.984
Height (cm)	162.21 ± 5.87	161.28 ± 5.88	0.351
Duration of anesthesia (min)	41.95 ± 8.23	42.64 ± 9.26	0.644
Duration of surgery (min)	37.90 ± 8.10	37.14 ± 8.66	0.594
Total amount of fluid (ml)	2264.28 ± 446.90	2227.14 ± 446.53	0.624
Duration of the delivery (min)	7.84 ± 2.93	8.17 ± 2.75	0.495
APGAR (1 min)	8.71 ± 1.10	8.68 ± 1.04	0.875

**Figure 2:**
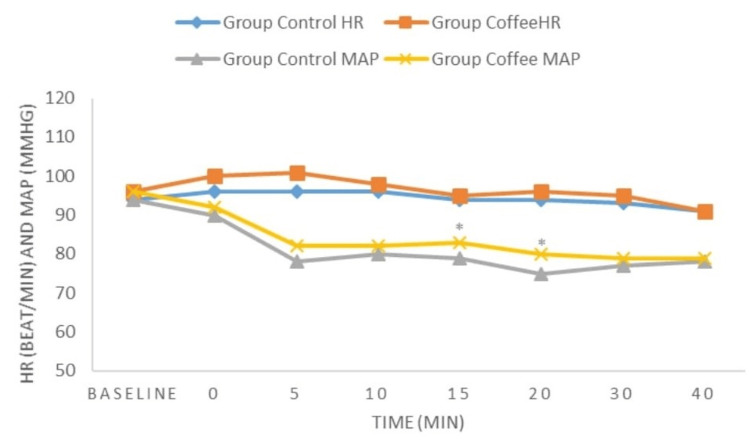
Comparison of heart rate (HR) and mean arterial pressure (MAP) values of the groups.

The incidence of hypotension and the rate of intraoperative ephedrine use were significantly lower in the coffee group (p = 0.006 and p = 0.001) (Table 2). There was no significant difference in heart rate values between the groups (p > 0.05). Mean arterial pressure values at the 15th and 20th minutes were significantly higher in the coffee group (p = 0.037 and p = 0.008) (Figure 2).

**Table 2:** Comparison of the incidence of hypotension and ephedrine requirement rates of the groups. Data are stated as number (n) and percentage (%). p < 0.05 according to the χ2 test.

Variables	Control group (n = 70)	Coffee group (n = 70)	p
Incidence of hypotension	50 (71.4%)	34 (48.6%)	0.006
Need of ephedrine	38 (54.3%)	18 (25.7%)	0.001

When the rates of nausea and vomiting were compared, there was no significant difference between the groups (p > 0.05). The rate of antiemetic use was found to be significantly lower in the coffee group (p = 0.016) (Table 3).

**Table 3:** Comparison of nausea and vomiting rates of the groups. Data are stated as number (n) and percentage (%). p < 0.05 according to the χ2 test. ** Fisher's exact test.

Variables	Control group (n = 70)	Coffee group (n = 70)	p
Rate of antiemetic use	15 (21.4%)	5 (7.1%)	0.016
Nausea	28 (40%)	19 (27.1%)	0.107
Vomiting**	5 (7.1%)	1 (1.4%)	0.104

When the PDPH incidences of the groups were compared, the coffee group had a significantly lower incidence of PDPH in the first 24 hours, while there was no significant difference between the PDPH incidences between 24 and 72 hours (p = 0.048 and p = 0.693). When the NRS values of the patients who developed PDPH were compared, there was no statistically significant difference between the groups (p > 0.05) (Table 4). None of the patients had PDPH persistent enough to require an epidural blood patch.

**Table 4:** Comparison of PDPH results of the groups. Data are stated as number (n), percentage (%), and mean ± SD. p < 0.05 according to the χ2 test and independent sample t-test. ** Fisher's exact test. PDPH: post-dural puncture headache; NRS: numerical rating scale.

Variables	Control group (n = 70)	Coffee group (n = 70)	p
PDPH, n (%) (first 24 hours)**	8 (11.4%)	2 (2.9%)	0.048
PDPH, NRS (mean ± SD) (first 24 hours)	4.12 ± 1.12	3.502 ± 0.70	0.486
PDPH, n (%) (24–72 hours)	16 (22.9%)	18 (25.7%)	0.693
PDPH, NRS (mean ± SD) (24–72 hours)	5.68 ± 1.66	5.55 ± 1.50	0.810

## Discussion

In this study, preoperative consumption of a single cup of coffee decreased the incidence of intraoperative hypotension and the rate of ephedrine use in cesarean section surgeries with spinal anesthesia. Also, the incidence of PDPH observed in the first 24 hours was lower in the coffee group.

Spinal anesthesia is a safe method of anesthesia in cesarean section. Hypotension is inevitable in 80-90% of cases as a common consequence of sympathetic block. Hypotension observed after spinal anesthesia might cause maternal and fetal adverse effects [12].

Vasopressors such as phenylephrine, which is an alpha-agonist, lead to peripheral vasoconstriction and an increase in systemic vascular resistance. Combined alpha and beta-agonists, such as ephedrine, can prevent hypotension by increasing both heart rate and systemic vascular resistance. Anaphylaxis, hypertension, and cardiac arrhythmias are possible adverse effects of vasopressors. They may also lead to impaired uteroplacental perfusion and fetal acidosis secondary to excessive vasoconstriction despite maintenance or restoration of maternal blood pressure. Ephedrine can develop tachyphylaxis and cause fetal acidosis [13].

A Cochrane analysis published in 2020 showed that anti-hypotension interventions such as crystalloids, colloids, ephedrine, phenylephrine, ondansetron, or lower leg compression could reduce the incidence of hypotension; however, none of these was sufficient to completely eliminate the need to treat maternal hypotension [13]. Another study reported that prophylactic ephedrine was more effective than Ringer's lactate and ondansetron [14]. Neither prophylactic drug administration nor intravenous fluid replacement were employed in this study. It is difficult to suggest that prophylactic coffee consumption is as effective as prophylactic vasopressor administration. However, coffee consumption did reduce the need for vasopressors. In this study, the incidence of hypotension (48.6%) was significantly lower in the coffee group. The incidence of hypotension in the control group was 71.4%. The results of this study showed that preoperative consumption of a single cup of coffee can reduce post-spinal hypotension without any side effects or complications. Coffee intake results in sympathetic activation, which is mediated by phosphodiesterase inhibition, cytosolic calcium increase, and stimulation of noradrenaline/adrenaline release [15]. The cardiac-stimulating effect of coffee seems to be effective in preventing the development of post-spinal hypotension.

Spinal anesthesia-related hypotension might cause distressing symptoms in the mother, such as nausea and vomiting. Uterine externalization, visceral stimulation, and uterotonic agents might also cause intraoperative nausea and vomiting [16]. Aksoy et al. [17] reported in their study that administration of granisetron and ondansetron before spinal anesthesia led to less need for ephedrine and less nausea and vomiting compared to the control group. In this study, a similar surgical manipulation was applied to all patients, and the same uterotonic agent was used; therefore, these factors were unlikely to affect the results of nausea and vomiting. Administration of antiemetics was performed in the presence of nausea and vomiting, which complicates the surgery and interferes with the patient’s comfort. The mechanism of post-spinal nausea and vomiting is complex. However, vagal mucosal tract induction in the gastrointestinal system is one of the causes. Therefore, the fact that coffee has a cardiac-stimulating effect by activating the sympathetic system suggests that it can be used to decrease the incidence of hypotension and nausea and vomiting due to hypotension.

PDPH is the most common postoperative complication of spinal anesthesia. Interventions leading to an improvement in headache relief can positively affect patients' satisfaction and decrease hospital days. Dexamethasone, pregabalin, and magnesium have been reported to reduce the incidence and severity of PDPH [18-20]. However, there has not been a sufficient number of studies conducted on the side effects of these agents, especially in infants.

There are two main pathophysiological mechanisms for PDPH. These are cerebrospinal fluid leakage leading to the traction of intracranial structures and compensatory vasodilation occurring due to intracranial hypotension, which causes headaches of the vascular type. The cerebral vasoconstriction and central nervous system stimulating effects of caffeine and coffee consumption have become a cornerstone of the treatment of PDPH [21]. In their study, Ragab et al. [21] reported that 500 mg of caffeine given intravenously in the first hour after spinal anesthesia reduces the incidence and severity of PDPH. Caffeine has been reported to be effective in the modulation of pain with the antagonism of adenosine receptors [22]. In this study, the incidence of PDPH, especially in the first 24 hours, decreased with the consumption of a single cup of coffee. Coffee consumption is non-invasive, does not cause any side effects at this dose, is cost-effective, and does not make the patient think that she is taking medication. Keeping the fetus in mind, a simple intervention like oral caffeine intake is a promising technique that can be used without any side effects. Coffee shows potential for clinical application.

Limitations

Ephedrine was the preferred vasopressor agent for this study since phenylephrine is not available in our country. In terms of PDPH, it might have been more accurate to monitor the patients for five to seven days instead of three days. The satisfaction and anxiety levels of the patients were not evaluated.

## Conclusions

In conclusion, the consumption of a single cup of coffee orally before spinal anesthesia reduced the incidence of intraoperative post-spinal hypotension, nausea, and vomiting, and the rate of ephedrine use. It also decreased the incidence of PDPH in the first 24 hours after surgery.
